# Supplementary material for: Serologic cross-reactivity of SARS-CoV-2 with endemic and seasonal Betacoronaviruses
Source: medRxiv. 2020 Jun 23:2020.06.22.20137695. Preprint. [Version 1] doi: 10.1101/2020.06.22.20137695 (PMC7315998; doi:10.1101/2020.06.22.20137695)
Supplement: Supplement 2020 [file 87061-2020.06.22.20137695-1.pdf]

SUPPLEMENTAL FIGURES AND LEGENDS

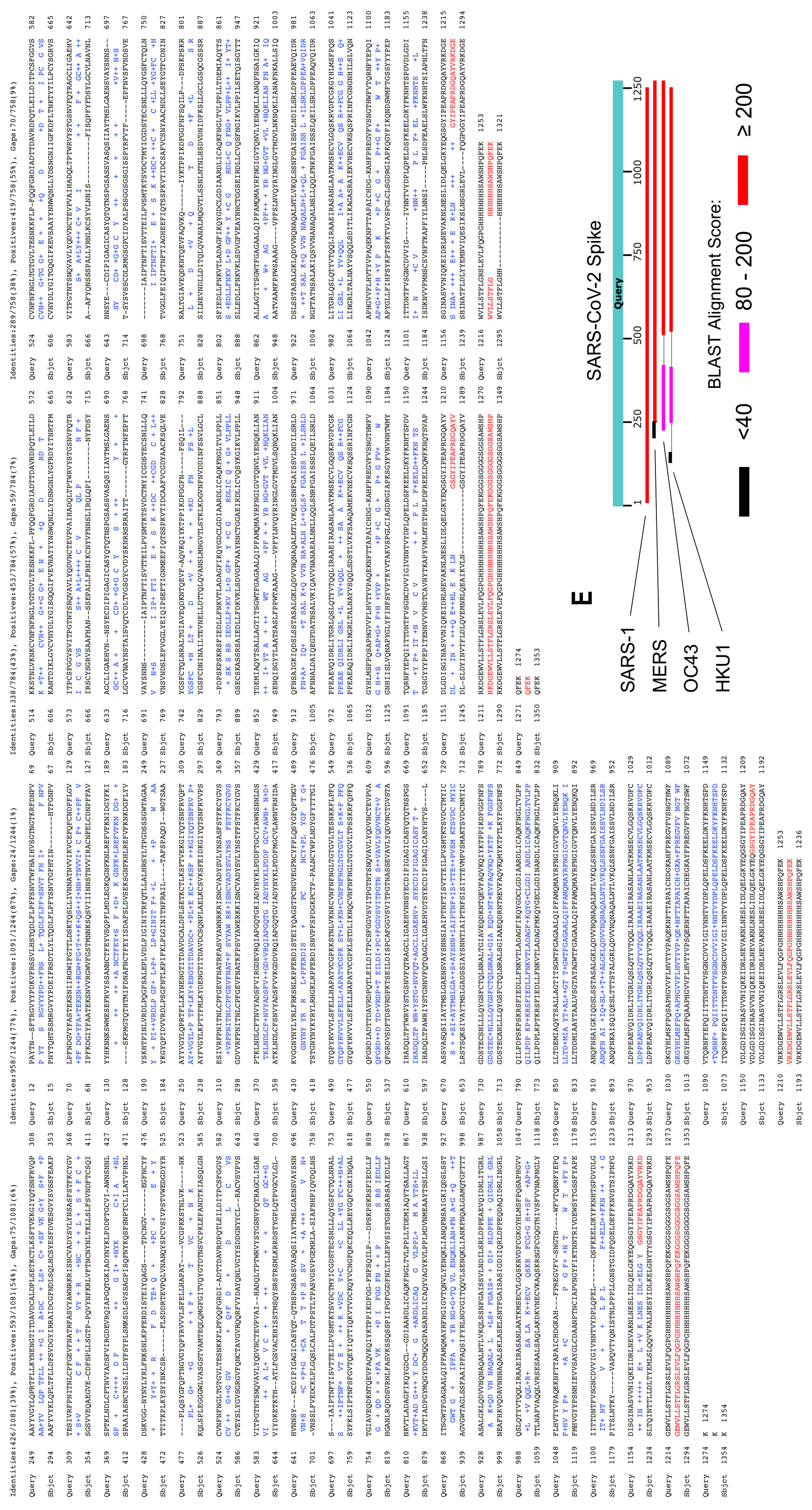

Supplementary Figure 1: BLAST alignment of 4 coronaviruses with SARS-CoV-2.

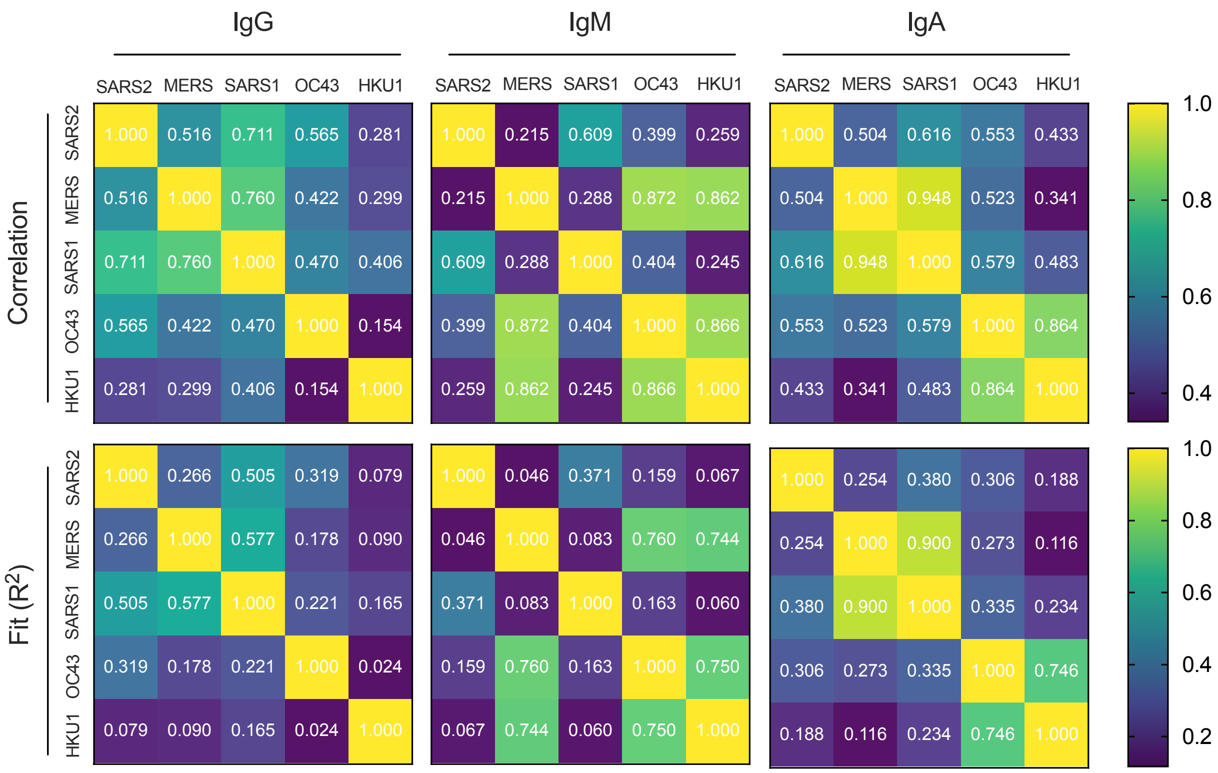

Supplementary Figure 2: Linear Correlation Statistics of *Betacoronaviruses*

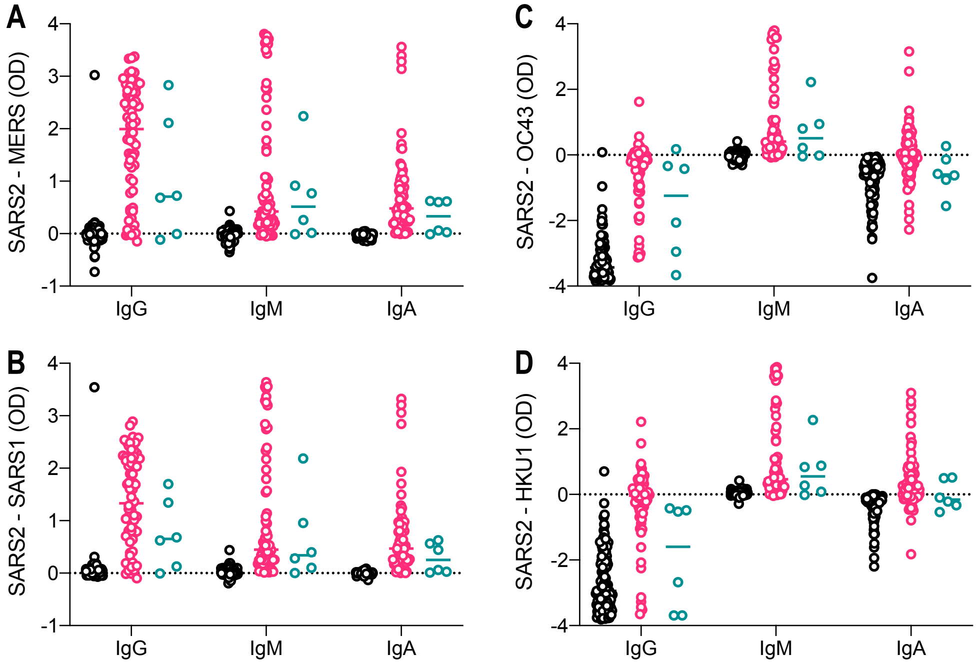

Supplementary Figure 3: Differential signal intensity of SARS-CoV-2 spike with other *Betacoronaviruses*. Signal intensity displayed as SARS-CoV-2 absorbance (A450 – A650) minus signal intensity of other *Betacoronaviruses*. (a) MERS, (b) SARS-CoV, (c) OC43, and (d) HKU1. Archival negative = black, pandemic hot-spot symptomatic = pink, pandemic hot-spot asymptomatic = teal. n =114 archival negative, n = 68 hot-spot symptomatic, n = 6 hot-spot asymptomatic.
